# Effects of COVID-19 on Multilingual Communication

**DOI:** 10.3389/fpsyg.2021.792042

**Published:** 2022-02-01

**Authors:** Maria Pilgun, Aleksei N. Raskhodchikov, Olga Koreneva Antonova

**Affiliations:** ^1^Interdisciplinary Research Laboratory, Russian State Social University, Moscow, Russia; ^2^Department of Psycholinguistics, Institute of Linguistics, Russian Academy of Sciences, Moscow, Russia; ^3^Moscow Centre of Urban Studies “Gorod”, Moscow, Russia; ^4^Department for Translation and Interpreting, University Pablo de Olavide, Seville, Spain

**Keywords:** speech perception, social media, COVID-19, neural network technologies, multilingual communication, vaccination

## Abstract

The paper presents the results of a study on the analysis of the perception of coronavirus disease 2019 (COVID-19) by Spanish-, German- and Russian-speaking social media actors after the emergence of vaccines and attitudes toward vaccination. The empirical base of the study was corpus data, materials from online media, social networks, microblogging, blogs, instant messengers, forums, reviews, and video hosting data. The Spanish-language database included 6,640,912 tokens and 43,251,900 characters; the German-language database included 16,322,042 tokens and 109,139,405 characters; and the Russian-language database included 16,310,307 tokens and 109,060,935 characters. With a neural network approach, a multilingual analysis was performed, which made it possible to analyze the topic structure and the semantic network with the allocation of the semantic core and the associative network. Differential and integral features of the identified structures based on the material of these three databases made it possible to determine the general and different characteristics of the perception by Spanish-, German-, and Russian-speaking users of the development of the pandemic, a number of social problems, attitudes toward various types of vaccines, observance of preventive measures, and readiness for vaccination.

## Introduction

Perception of decease: The cognitive representation of a disease present in both patients and healthy individuals largely determines the emotional and behavioral responses of members of society. The specificity of the disease perception is one of the important factors in decision-making within the system of preventive measures, transformation of the health care system, as well as social, political, and economic spheres, especially during a pandemic.

A note should be made of the coronavirus disease 2019 (COVID-19) IMPACT project^1^, which is an international online survey conducted in 78 countries/regions of the world aimed at studying behavioral and psychological consequences of COVID-19. Based on the project data, a study was conducted in 16 European countries in the early period of the pandemic, which showed that Europeans reacted in a similar way to information about the COVID-19 spread in other countries; national differences were of no importance, but age, gender, and educational background affected the perception of COVID-19 under certain conditions. In addition, perceptions of this decease were more consistent in explaining overall stress than taking preventive measures against COVID-19 (Dias Neto et al., 2021). The perception of COVID-19, the reaction of society to the introduced anti-COVID-19 measures, the need for protective measures (the requirement to observe social distancing, wear masks, wash hands, etc.) largely determines the behavior of people and, as a result, the effectiveness of the fight against the pandemic (Bilgili et al., 2021).

In the absence of a vaccine or treatment for COVID-19, all measures to contain and limit the spread of the infection depend on the behavior of the people. Meanwhile, since the beginning of the pandemic, 2 types of responses to the infection spread have been formed: one part of society perceived the new infection with anxiety and even fear and was actively engaged in preventive protection; the other part, on the contrary, ignored the danger of the disease and protested sharply against all introduced preventive measures (Bump, 2020; Burnett, 2020; Dave et al., 2020; Malone and Bourassa, 2020; Niemi et al., 2021; Tagini et al., 2021).

During the COVID-19 pandemic, the World Health Organization introduced the concept of “Infodemia” to show the danger to global society, in the age of social networks, the distortion of reality in the rumble of echoes and comments of the global community on real or often invented facts. Thorough studies have already been carried out on how the coronavirus situation is described from journalistic communication (Papapicco, 2020).

For example, COVID-19 threat assessment, trust in information sources, and fear of the infection are important predictors of COVID-19 preventive behavior in Latvian citizens. Beliefs of a COVID-19 conspiracy significantly reduce the threat assessment of the new disease and the credibility of relevant information, and the fear of COVID-19 is determined by threat assessment, which is the most important factor associated with COVID-19 preventive behavior (Šuriņa et al., 2021).

Research has also shown that Americans perceive the COVID-19 threat as significantly greater than other causes of death to which it has recently been compared, including seasonal flu and car accidents. It is important to note that citizens were less apt to help victims of COVID-19, as they consider such assistance to be dangerous for themselves, and patients with COVID-19 to be more infectious and more responsible for their condition (Niemi et al., 2021). Similar conclusions are drawn by other researchers who have found that many people, such as health care providers and citizens living in high-risk areas, may experience negative attitudes caused by COVID-19 (Adja et al., 2020; Singh and Subedi, 2020). Patients with COVID-19 have experienced various types of discrimination, such as isolation, denial of service, harassment, and bullying (Turner-Musa et al., 2020). As a result, people who become infected or only suspect they have symptoms of COVID-19 postpone seeking medical help or even hide the disease. Such behavior seriously threatens the safety of others, makes it extremely difficult to take anti-COVID-19 measures, and contributes to the spread of the infection (Dubey et al., 2020). Discrimination against patients with COVID-19, according to experts, is widespread throughout the world, for example, in America, Nepal, Jordan, India, Italy, and China (Aacharya and Shah, 2020; Chopra and Arora, 2020; Khasawneh et al., 2020; Sahoo et al., 2020; Singh and Subedi, 2020; Turner-Musa et al., 2020; Zhao et al., 2021a).

The psychological problems caused by COVID-19 have also received coverage from various countries (Al-Omiri et al., 2021; Zhao et al., 2021b).

The emergence of vaccines marked a new stage in the pandemic; the focus on the analysis of COVID-19 perception was naturally changed to the analysis of society’s readiness to get vaccinated actively. Since COVID-19 vaccination is voluntary, everyone’s willingness to participate in vaccination campaigns is the key factor in the success of pandemic control (Lu et al., 2021).

Attitudes toward COVID-19 vaccines have begun to be actively studied in various populations in various countries, since it is obvious that, for effective vaccination of the population, it is important to increase public confidence and awareness of the efficacy and safety of COVID-19 vaccines. Simulations have shown that vaccination of older people reduces deaths, while vaccination of younger and more socially active people minimizes infections (Wang et al., 2021). For example, researchers found that Chinese teenagers have a positive attitude toward COVID-19 vaccines (Cai et al., 2021). Analysis of the intention to receive a vaccine against COVID-19, as well as predictors of such intentions in the Norwegian population, showed that the majority (61.6%) of participants intend to get vaccinated, 24.8% of the population are not sure, and 13.8% are not going to receive the vaccine (Wolff, 2021). It is important to analyze the motivation of citizens of various countries when making a decision on vaccination. Researchers found that, among Americans, older Asian men with higher levels of education correlated with vaccination (Malik et al., 2020). Predictors of a positive vaccination decision for British adults are positive beliefs, less fear of side effects, willingness and positive attitude toward obtaining the necessary information to make a reasonable decision to get vaccinated against COVID-19, an increased perception of the COVID-19 risk to others (but not to themselves), old age, and participation in the influenza vaccination in winter 2019/20 (Sherman et al., 2020). For Australians, refusal to get vaccinated is determined by the belief that the COVID-19 threat is exaggerated by inadequate medical literacy and under education (Dodd et al., 2021). North American respondents prefer to rely on natural immunity; the lack of confidence in the benefits of the vaccine is determined by fears of unintended consequences in the future and unwillingness to contribute to the commercial profit of pharmaceutical companies (Rosman et al., 2021). Results of German studies show that, since April 2020, when the intention to get vaccinated was estimated at about 79%, a steady decline has been observed throughout 2020. The lowest rates were recorded in early and mid-December, when only about 48% of the population were ready to get the vaccine against COVID-19. Following this drop, support for vaccination has risen again to 68% by early March 2021 (Betsch et al., 2021).

Significantly, public opinion about experience and credibility is critical to the success of a vaccination campaign. Trusting scientists and public health experts who make informed claims about COVID-19 and about the safety and efficacy of vaccines is essential to a successful vaccination campaign. If the public no longer believes the official expertise, then it becomes impossible to control the pandemic. Vaccinations, testing, masking requirements, non-drug interventions are compromised. In such a situation, the importance of effective crisis communication increases (Rosman et al., 2021).

Yanni Zhang, Naveed Akhtar, Qamar Farooq, Yiwei Yuan, and Irfan Ullah Khan conducted a critical discourse analysis aimed at exploring the dialectical relationship between discourse and ideology to reveal hidden psychological messages and ideology in the informational coverage of the pandemic. Psycholinguistic techniques, news reports, and comments from Chinese and American media about COVID-19 were analyzed, and the authors used Wang Zhenhua’s Appraisal Theory and Halliday’s Systemic Functional Grammar as tools to make a comparative analysis of the corpus (Zhang et al., 2021).

The purpose of this study was to identify the characteristics of the perception of the COVID-19 pandemic by Spanish-, German-, and Russian-speaking social media participants after the emergence of vaccines and the attitude toward vaccination itself.

Germany, Russia, and Spain are among the countries most severely affected by the coronavirus; however, all three countries belong to different types of cultures (Hofstede, 2015), which makes a comparative study of the response of society to the crisis situation caused by the pandemic especially interesting.

The COVID-19 pandemic has transformed almost all spheres of society, transferred communication processes to the virtual space, which enhances the importance of studying digital data.

Studies that were previously conducted in various countries to analyze the perception of the coronavirus infection and readiness for vaccination were mainly based on survey data; however, social media materials using psycholinguistic methods and neural network technologies have not yet been applied.

## Materials and Methods

### Data Collection

To collect data, Sketch Engine^2^ systems were used.

In accordance with the specifics of the program, the data were collected according to several lists. After the selection of the material, all data were combined into one database for each language:

• Covid, vaccine, vaccination, EpiVacCorona, CoviVac, Sputnik, Sputnik V, Sputnik Light, Moderna, AstraZeneca, Pfizer, and Biontech.
• Risk group, immunity, covid dissidents, vaccination, covidiots, placebo, injections, side effect, antibodies, contraindications, Gamaleya Center, Chumakov Center, and Vector.
• Vaccinated, anti-masker, certificate of a vaccinated person, certificate of vaccination, anti-vaxers, anti-vaccinators.
• Collective immunity, agitation, those who had recovered, come through, infection, infect, and prevent.
• Protection, freedom, choice, quarantine, wave, strain, and variant.

### Data

The empirical base of the study was corpus data, materials from online media, social networks, microblogging, blogs, instant messengers, forums, reviews, and video hosting data.

Quantitative characteristics of the data:

The Spanish-language database included 6,640,912 tokens and 43,251,900 characters.
The German-language database included 16,322,042 tokens and 109,139,405 characters.
The Russian-language database included 16,310,307 tokens and 109,060,935 characters.

### Methods

To analyze the content of social media, a multimodal approach was used using neural network technologies, text analysis, content analysis, sentiment analysis, and analysis of lexical associations.

The research used experience of content analysis technologies, including Sketch Engine and other quantitative automated systems and qualitative manuals presented in Papapicco and Mininni (2020).

With a neural network approach, a multilingual analysis was performed, which made it possible to identify the topic structure and the semantic network with the identification and analysis of its semantic core and associative network.

NLP topic modeling and clustering techniques are used to catalog, analyze, and automatically extract topics from datasets, such as survey responses. For example, topic modeling can use the identification of groups of words that often occur together (Lossio-Ventura et al., 2021), and clustering methods help group texts based on their similarity. The advantages of the neural network approach for topic modeling became apparent after the appearance of the BERT language model (Google) (Zhou et al., 2019; Liu et al., 2020; Zhao et al., 2021a,b). In particular, BERT enhances the semantic representation of texts with its feature extraction and fine-tuning transfer learning capabilities (Vaswani et al., 2017; Devlin et al., 2019; Lossio-Ventura et al., 2021).

In this study, the neural network technology TextAnalyst 2.3 was used for multilingual analysis of user perception. This model allows for automatic semantic ranking of the textual database using several algorithms: an algorithm for forming a homogeneous frequency network of text using an artificial neural network based on neural-like elements with time summation of signals, and an iterative Hopfield-like algorithm for ranking network vertices on a scale of 0–100%. In addition, the n-gram representation of the network is formed by iterative re-weighting at a given number of steps, or based on the convergence criterion of the ranking process. Thus, lexemes are analyzed in the context of syntagmas of a given (n) length on a semantic network formed on the basis of text analysis. The frequency network of the text is built as a set of pairs of words that are found in the sentences of the text. The network vertices are weighted by their frequency of occurrence in the text. The connection weight of a pair of vertices in the network corresponds to the frequency of occurrence of word pairs in text sentences (Kharlamov and Pilgun, 2020).

After identification of a topic structure, a semantic network was formed, in which semantic clusters were identified and analyzed.

Of particular importance for the analysis of the actors’ perception is the analysis of lexical associations. An associative search was performed, associative networks were built, and reactions to similar stimuli in the three analyzed language bases were analyzed.

Word Association (WA) paradigms are applied across various types of research (Brooks et al., 2014; Vivas et al., 2019). With the help of Implicit Association Tests (IATs), implicit social cognition, subconscious motivations, attitudes toward the presented stimulus, as well as automatic associations for subjects that hide at a conscious level (see, for example, Project Implicit)^3^ are studied. The potential of associations in the analysis of various types of network data has also already shown its effectiveness (File et al., 2019). In this study, a multilingual associative search was used, which allowed building relevant associative networks for similar stimuli in different language databases (Kharlamov and Pilgun, 2020). Thus, based on the material of the Spanish, German, and Russian datasets, the reactions of actors were identified and analyzed, which made it possible to draw conclusions about the preferential perception of users, to highlight and analyze the most frequent associations, peculiarities of perception of the COVID-19 pandemic by Spanish-, German-, and Russian-speaking social media participants after the emergence of vaccines. Also, a comparative analysis was performed, and ways of conducting predictive analytics were outlined.

### Tools

To collect data, Sketch Engine (see text footnote 2) systems were used.

The verbal content was analyzed using the neural network technology TextAnalyst 2.3^4^.

Content analysis was performed using the AutoMap text mining tool^5^.

For visual analytics, the Tableau platform was used^6^.

## Results

### Spanish-Speaking Actors

#### Topic Structure

Explicitly expressed information that makes up the topic structure of the Spanish database makes it possible to identify the following topics that were of greatest interest for users when they discussed the problems of COVID-19 after the emergence of vaccines:

• Effect of the vaccine on the **population** (*población*/connection weight—80):

*En el caso de México, la tasa de letalidad bruta derivada de una infección por la COVID-19 ha alcanzado cifrascercanas al 11%, para situarse entre uno de los países cuya población, una vez contagiada por esta enfermedad, tiene unamayor probabilidad de fallecer.*

• Features of various vaccines and their efficacy (**Pfizer:** connection weight—82, AstraZeneca—81):

*La mayoría de los inmunizados ha recibido el preparado de Pfizer (17.956.122), seguido de Moderna (2.176.152), AstraZeneca (4.503.479) y la monodosis de Janssen (1.784.344).*

• Vaccination duration (**week/*semana*–** Bec связи–85; **hours/*horas***: connection weight—89; frequency—19, 02; **month/*mes***—74, **day/*día***: connection weight—89):

*En cuanto a grupos de edad, casi el 90% de los españoles de 60 a 69 años tienen la pauta completa de la vacuna, en su mayoría de AstraZeneca, que requiere más tiempo de intervalo entre dosis, entre 12 y 14 semanas, motivo por el que varias comunidades adelantaron la segunda dosis para hacer frente a la expansión de la variante delta.*

• Significance and features of **vaccination (*vacunación***: connection weight—86):

*Carolina Darias, ministra de Sanidad: “España se convierte en el segundo país de la Unión Europea con el porcentaje de vacunación completa más alta.”*

• Effect of vaccination on the spread of **COVID-19** (connection weight—92):

*El número de muertes por COVID-19 ha sido 15, lo que supone un descenso con respecto a ayer, cuando se registraron 24.*

• Factor for priority vaccination—age (años/years—69):

*En cuanto al plan de vacunación, la Comunidad de Madrid ha administrado 7.564.441 dosis, sobre un total de 7.903.484 recibidas y el 52,5% de la población general ha recibido la pauta completa, cifra que alcanza al 63,4% de la población diana (mayores de 16 años).*

• New cases, spread of the infection and fear of the disease (***contagio*/contagion**—81, enfermedad/disease—82):

*La Comunidad de Madrid ha notificado 5.167 nuevos contagios, de los que 3.523 se diagnosticaron en las últimas 24 horas, y siete fallecidos (uno más que ayer), al igual que sigue aumentando la presión hospitalaria.*

#### Semantic Network

**FIGURE 1:**
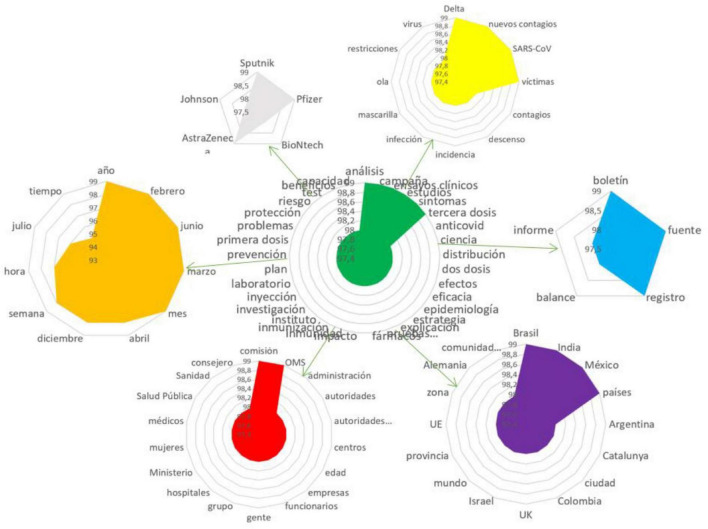
Features of vaccine production and specificity of vaccination.

The identification of the semantic network and the analysis of the semantic core make it possible to study the semantic accents that users focus on when discussing the analyzed problem; semantic clusters with implicit information are indicative of the users’ opinions and assessments (Figure 1):

Significant nominations of the semantic network with connection weights of (99): analysis, vaccination campaign, science, clinical trials, symptoms, third dose, anti-COVID-19 measures, 98 vaccine distributions, first dose, two doses, effect, efficacy, tests, risk, strategy, explanations, pharmaceutical research, result, immunity, safety, laboratory, plan, prevention, problems, protection, benefits, intensity, and distribution.

Contexts are as follows:

*Insiste el inmunólogo español en este mensaje. «Aunque los anticuerpos disminuyan, gracias la memoria inmunológica, cuando entremos en contacto con el virus, se van a activar y van a generar una respuesta». Entonces, ¿hace falta una tercera dosis de refuerzo?*

1. Discussion of various types of vaccines.

Significant nominations of the semantic network with connection weights of (99): Pfizer, BioNtech (98), AstraZeneca, Johnson, Jansson, and Sputnik.

Contexts are as follows:

*“Según criterio del especialista, pueden recibir cualquiera de las dosis disponibles ya sea a virus inactivado, de vector viral, o ARN mensajero. Entonces, cualquiera de las autorizadas en el país como Sputnik, Sinopharm o AstraZeneca pueden ser administradas en este tipo de huéspedes,” indica.*

2. Spread of infection.

Significant nominations of the semantic network with connection weights of 99: delta variant, SARS-CoV, new infections, victims; 98—infection, recession, incidence, infection, masks, wave, restrictions, and virus.

Contexts are as follows:

*Los datos sobre la situación epidemiológica en España y gran parte del mundo no son demasiado alentadores. En nuestro país ayer la incidencia alcanzó los 644 casos por 100.000 habitantes, más de 30.500 contagios en 24 horas y todavía se espera al pico de la quinta ola, que en Catalunya parece que ya alcanzó recientemente, dando pie al descenso.*

3. Territorial features of the infection spread and vaccination:

Significant nominations of the semantic network with connection weights of 99: Brazil, India, Mexico, countries, Europe, EU, region, 98—Argentina, Catalonia, city, Colombia, Israel, world, province, zone, Germany, Great Britain, and autonomous communities.

Contexts are as follows:

*Laboratorios de Biológicos y Reactivos de México (Birmex) anunció el 7 de mayo que a partir de finales de junio iniciaría con el proceso para envasar la vacuna rusa Sputnik V en México. Sin embargo, los reactivos para envasar llegaron a México apenas este miércoles.*

4. Subjects of vaccination, persons responsible for vaccination.

Significant nominations of the semantic network with connection weights of 99: commission, WHO, 98—administration, government entities, medical institutions, medical centers, age groups, companies, public sector employees, people, groups, hospitals, ministry, women, doctors, health care, and advisers.

Contexts are as follows:

*El lunes 12 de julio arranca en España la fase III del estudio multicéntrico mundial que Pfizer y BioNTech está llevando a cabo para evaluar la seguridad y eficacia de su vacuna en mujeres embarazadas.*

5. Time characteristics of vaccination.

Significant nominations of the semantic network with connection weights of 99: year, February, June, month, 98—April, December, week, hour, day, time, etc.

Contexts are as follows:

*Los enfermos de covid persistente llevan muchos meses, algunos incluso más de un año, sufriendo la sintomatología covid prácticamente a diario. Por edad—el perfil medio de estos pacientes es el de una mujer de 43 años, aunque también los hombres sufren esta patologí a-, muchos de ellos han añadido recientemente una preocupación más a su dura cotidianidad: los posibles efectos de la vacuna.*

6. Information support of vaccination.

Significant nominations of the semantic network with connection weights of 99: record, source, register, and report.

Contexts are as follows:

*La vacuna es segura. Todas las vacunas aprobadas son sometidas a pruebas rigurosas a lo largo de las diferentes fases de los ensayos clínicos, y siguen siendo evaluadas regularmente una vez comercializadas. Los científicos también siguen constantemente la información procedente de diferentes fuentes en busca de indicios de que una vacuna pueda tener efectos adversos.*

The analysis of the Spanish semantic network enables identification of semantic accents that are of particular importance for actors, characterize the perception of the vaccination process, its protection and risks, as well as the principle of its distribution, which in itself already implies agreement with vaccination, even considering the necessity of the third dose. When discussing the specifics of the vaccine production and the vaccination process, actors focus on issues of vaccine knowledge, number of doses, immune response, explanatory process and vaccination strategies. After the emergence of several types of vaccines, actors began to actively discuss the merits of specific vaccines. Pfizer/BioNtech, AstraZeneka, Janssen, and Sputnik received the most attention in the discussion. All vaccines are of foreign origin. Despite the fact that the vaccination process has begun, Spanish users continue to worry about the spread of the infection, its new variants, and waves of infections; at the same time, actors emphasize the decline in the spread of the infection. In addition, other anti-infection measures such as masks and other restrictions are still in place. In the segment of the semantic network dedicated to persons responsible for vaccination, doctors, communities, risk groups, government entities, women, and public sector employees take pride of place. In the vaccination process, Spain aligns itself with the WHO, as well as with the experience of various EU countries and other Latin American countries because of the language proximity, and organizes the process on a territorial basis, while participation in vaccination differs in different cities and autonomous communities.

The actors are actively discussing the time characteristics of vaccination, age factors, as well as the timing required to test a new vaccine and identify *side effects*.

Information support for vaccination, according to Spanish-speaking actors, as confirmed by the semantic network data, is very scarce and limited to records and reports.

#### Associative Network

The analysis of lexical associations based on the results of the associative search and the associative network built made it possible to identify implicatures, subtextual information characterizing the attitude of actors to vaccination, peculiarities of actors’ perceptions of the COVID-19 pandemic after the commencement of vaccination, of various processes associated with the creation of vaccines, and the organization of the vaccination process.

##### Stimulus Pfizer

**FIGURE 2:**
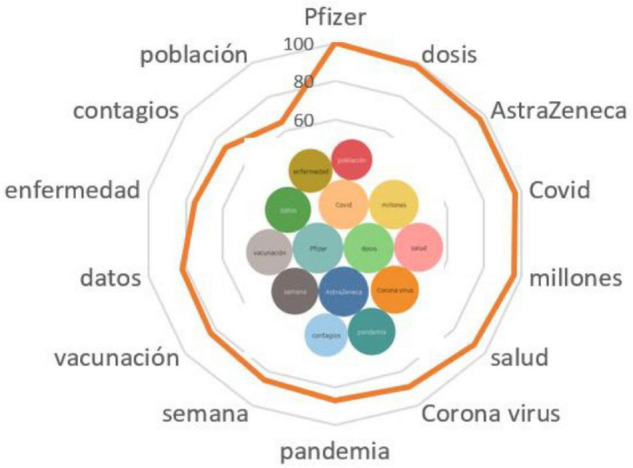
Associative network of the stimulus *Pfizer.*

Contexts with responses:

*La vacuna de Pfizer utiliza ácido ribonucleico mensajero; es una tecnología nueva para vacunas pero tenemos muchos años investigándolo -desde hace 30 años- como candidatos a medicinas. Es una vacuna muy eficaz que empieza a proteger desde la primera dosis y protege muy alto luego de la segunda dosis* (Figure 2).

As part of the fight against the pandemic and the arrangement for batch manufacturing of the Pfizer vaccine, Spanish-speaking actors pinned their great hopes on improving the health of their fellow citizens, on reducing the number of infected with each dose received (*pandemia, enfermedad, salud, contagios, población, vacunación, corona virus, millones, dosis*). The effect stretched for weeks; users began to compare the efficacy of the Pfizer vaccine with other vaccines used in Spain, such as AstraZeneca.

##### Stimulus Risk Group/Grupo de Riesgo

**FIGURE 3:**
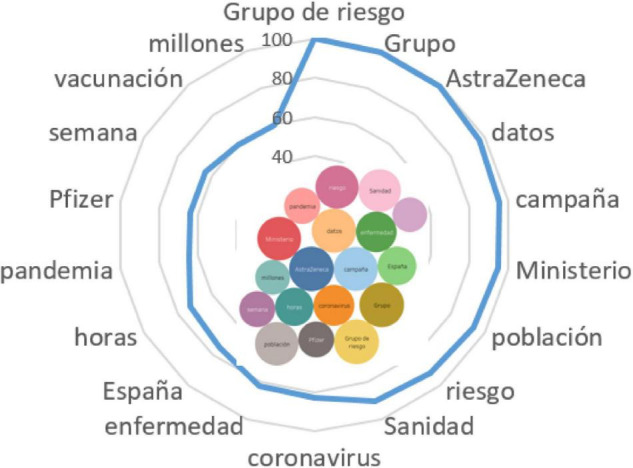
Associative network of the stimulus risk group/*Grupo de riesgo.*

Contexts with responses:

*En este grupo, hay muchas dudas sobre la inmunización. Las respuestas de los especialistas a las preguntas clave. Los pacientes oncológicos están en el grupo de riesgo para recibir la vacuna* (Figure 3).

The analysis of the associative network also made it possible to conclude that the Spanish-speaking actors trust their healthcare community (*Sanidad, Ministerio*). In addition, the Spanish actively discussed and worried about the situation with risk groups during the pandemic (*grupo de riesgo, pandemia*). The vaccination campaign in Spain implies the use of *AstraZeneca* and *Pfizer* vaccines, which are supposed to prevent the spread of diseases caused by the coronavirus infection (*enfermedad, coronavirus, población, compaña*). Actors believe that the existing data make it possible to organize mass vaccination of risk groups as well, provided that the protocol is strictly adhered to (*datos, semanas*).

### German-Speaking Actors

#### Topic Structure

Explicitly expressed information that makes up the topic structure of the German database makes it possible to identify the following topics that are of greatest interest for users discussing the problems of COVID-19 after the commencement of the vaccination campaign:

• Effect of the vaccine on the **population** (*Menschen*/connection weight—74, persons/*Personen*—64):

*Obwohl ein milder Verlauf der Krankheitinsbesondere bei jungen Menschen häufig ist und die meisten Erkrankten vollständig genesen, sind schwere Verläufe mitLungenentzündung, die über ein Lungenversagen zum Tod führen können, möglich.*

• Features of various vaccines and their efficacy (**vaccine/*Impfstoff***: connection weight—74):

*Der hier besprochene Vektor-Impfstoff (COVID-19 VACCINE Janssen) ist ein gentechnisch hergestellter Impfstoff.*

• Significance and features of **vaccination (*Impfung***: connection weight—72):

*Es gibt keine spezifische Therapie. Neben dem Vermeiden einerInfektion durch Beachtung der AHA* + *A* + *L-Regeln (Abstand halten, Hygiene beachten, Alltagsmaske tragen, Corona-Warn-Appherunterladen, regelmäßig lüften) bietet die Impfung den bestmöglichen Schutz vor einer Erkrankung.*

• Effect of vaccination on the spread of **COVID-19** (connection weight—75, *Corona*—76):

*Die Genspeed Biotech GmbH in Rainbach im Mühlkreis (Bezirk Freistadt) stellt einen weltweit einzigartigen COVID-19-Schnelltest her, der Antikörper gegen das Virus nach einer Erkrankung nachweist und in einer weiteren Ausbaustufe sogar eine aktuelle Infektion belegen soll.*

• Pronounced territorial factor of vaccination—Germany (*Deutschland*—69):

*Mittlerweile wird auch in Deutschland eine sogenannte “Kreuzimpfung,” also eine Corona-Impfung aus zwei Impfstoffen, angeboten.*

#### Semantic Network

**FIGURE 4:**
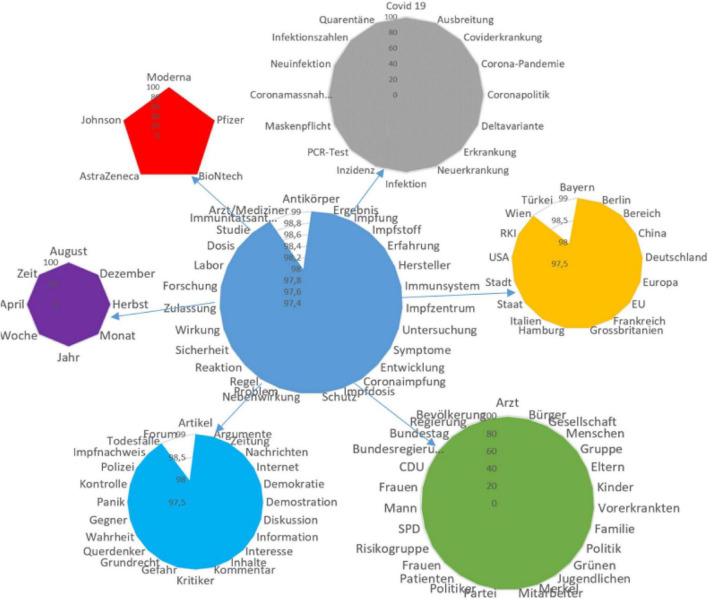
Features of vaccine production and specificity of vaccination.

Significant nominations of the semantic network with connection weights of (99): *antibodies, doctor (98), examination, vaccine, development, result, experience, production, immune system, vaccination, dose, vaccination center, protection, side effects, problems, rules, reaction, safety, symptoms, action, access, research, vaccination commission, laboratory, and immune response* (Figure 4).

Contexts are as follows:

*Doch ab 14 Tagen nach der zweiten Dosis waren die T-Zell-Antworten in beiden Gruppen vergleichbar. Allerdings ist ebenfalls noch nicht klar, ob die Antikörper- oder die T-Zell-Antwort wichtiger für den Schutz gegen COVID-19 sind*—*oder ob beide gleichermaβen eine Rolle spielen.*

7. Discussion of various types of vaccines.

Significant nominations of the semantic network with connection weights of (99): *Moderna, Pfizer, BioNtech, AstraZeneca*, and *Johnson*.

Contexts are as follows:

*Biontech, Moderna, and AstraZeneca: Wie es gegen Mutanten helfen könnte, Impfstoffe zu kombinieren.*

8. Spread of infection.

Significant nominations of the semantic network with connection weights of 99: *covid, spread, pandemic, covid policy, delta strain, disease, new infections, infection, inditions, masks, PCR test, number of cases, and quarantine*.

Contexts are as follows:

*Studien aus Israel und Grossbritannien legen auch nahe, dass vor allem Biontech Ansteckungen deutlich vermindert.*

9. Territorial features of the infection spread and vaccination.

Significant nominations of the semantic network with connection weights of 99: *Bavaria, Berlin, China, Germany, Europe, EU, France, United Kingdom, Hamburg, Italy, Switzerland, state, city, United States, Vienna, and Turkey* (98).

Contexts are as follows:

*Darüber hinaus haben drei Impfstoffe aus China eine Notfallzulassung im eigenen Land erhalten, obwohl die Phase-3-Studien noch nicht abgeschlossen waren.*

10. Subjects of vaccination, persons responsible for vaccination.

Significant nominations of the semantic network with connection weights of 99: *doctor, citizens, society, people, group, parents, children, people with pathologies, family, party, Green Party, youth, Merkel, employees, patients, women, politicians, risk groups, Socialist Party of Germany, man, federal government, Bundestag, government, population, and CDU party*.

Contexts are as follows:

*12.45 Uhr: SPD-Gesundheitsexperte Karl Lauterbach hält die Delta-Variante für deutlich gefährlicher als bisherige Mutanten des Coronavirus.*

11. Time characteristics of vaccination.

Significant nominations of the semantic network with connection weights of 99: *year, time, day, week, month, autumn, August, December*, etc.

Contexts are as follows:

*Demnach ist der Impfstoff von Pfizer/Biontech zwei Wochen nach der zweiten Dosis zu 88 Prozent wirksam gegen eine durch die Delta-Variante ausgelöste COVID-19-Erkrankung, bei der Alpha-Variante sind es 93 Prozent.*

12. Information support of vaccination.

An information campaign to support vaccination faced criticism and protests from German-speaking actors, who, focusing on their rights, insisted on their right to choose. Significant nominations of the semantic network with connection weights of 99: *article, forum, newspaper, arguments, YouTube, news, internet, democracy, demonstration of protest, panic, control, police, discussion, information, interest, content, comment, critic, danger, constitutionally eligible, negacionists, truth, freedom, and anti-vaccination*.

Contexts are as follows:

*PIMS ist für mich dazu kein Argument, vor allem weil man es—wie viele andere Dinge zu Corona—einfach als etwas völlig neues in den Raum geschmissen hat, obwohl dieses sehr seltene Phänomen auch schon vor Corona vorhanden war, oft dann aber im Zusammenhang mit dem Kawasaki-Syndrom in Zusammenhang gebracht.*

The analysis of the German semantic network makes it possible to identify semantic accents that are of particular importance for actors and characterize the perception of the vaccination process and the motivation for making a decision whether to get vaccinated. When discussing the specifics of the vaccine production and the vaccination process, the actors highlight the issues of vaccine knowledge, vaccination rules, immune system response, and number of doses. After the emergence of several types of vaccines, the actors began to actively discuss the merits of specific vaccines. Moderna, Pfizer/BioNtech, AstraZeneka, and Johnson received the most attention in the discussion, and Germany also participated in the production of some of them. Despite the vaccination, people in Germany are worried about the spread of infection, its new variants, serious consequences, and deaths from the coronavirus infection. Moreover, other protective measures against infection are still in place, such as tests, quarantine, self-isolation, and wearing masks. In the semantic web, a segment is distinguished associated with those responsible for administering vaccinations, along with communities, population groups, with different gender and professional characteristics. The actors are actively discussing the actions of the authorities, politicians, and leading political parties in Germany on the eve of the elections. In the vaccination process, Germany is guided by its own research institute RKI, the experience of various EU countries and other countries, and also organizes vaccination on a territorial basis, and the organization of vaccination differs in individual cities and provinces.

The actors are actively discussing the timing of vaccination, age factors, as well as the time required to test a new vaccine and identify *side effects*.

The informational support of vaccination according to the semantic network has a pronounced critical connotation. There is a clear protest against compulsory vaccination; demands are made to provide truthful information about the risks of vaccines, respect for democratic foundations, freedom of choice and demonstration of will, and protection against discrimination against vaccine opponents.

#### Associative Network

##### Stimulus BioNTech

**FIGURE 5:**
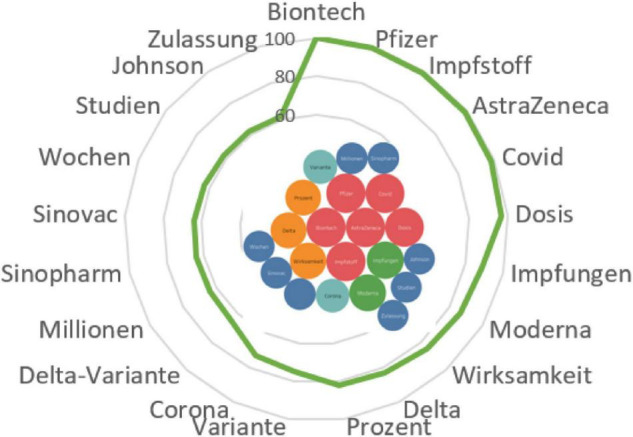
Associative network of the stimulus *BioNTech.*

Contexts are as follows:

*Bei Biontech berichten Geimpfte das Gegenteil: Hier fallen die Impf-Nebenwirkungen nach der zweiten Impfung stärker aus als nach der ersten* (Figure 5).

The BioNTech/Pfizer vaccine jointly developed by Germany and United States is actively discussed by German actors who compare it with other vaccines: AstraZeneca, Johnson, and Moderna. Users are concerned about its efficacy (*Wirksamkeit, Prozent*) against the coronavirus, as well as against the new Delta strain (*Variante Delta*). Along with familiarization with the protocol of its administration (*Wochen*), the reliability of research (*Studien*) and the availability of official permission for mass production (*Millonen, Zulassung*), its alternatives are also being considered, namely Sinopharm and Sinovac. Evidence shows that German-speaking citizens are taking vaccinations thoroughly by studying the situation and weighing other options.

##### Stimulus Risk Group (Risikogruppe)

**FIGURE 6:**
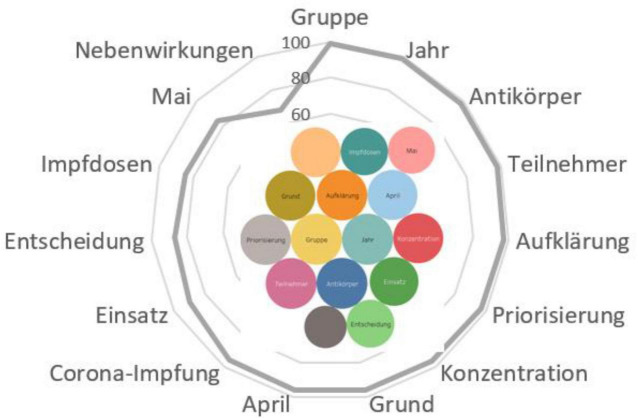
Associative network of the stimulus *risk group (Risikogruppe).*

Contexts with responses:

*Die Infektionskrankheit COVID-19 kann einigen Menschen sehr gefährlich werden. Besonders riskant ist eine Corona-Infektion für Ältere, chronisch Kranke und Menschen mit einem geschwächten Immunsystem. Zu dieser Risikogruppe zählen auch Krebskranke, etwa Männer mit Prostatakrebs* (Figure 6).

For German-speaking actors, the risk group includes primarily elderly people (*Jahre, Teilnehmer*), who are given priority in the vaccination process (*Corona-Impfung, Dosis, Priorisierung*). The actors believe it is necessary in such cases to carefully study the dose of the vaccine and the reasons for including citizens into the risk group, to calculate the concentration of antibodies, and to prevent side effects. German users place great emphasis on the need for outreach to ensure that vaccination decisions are well-thought-out and the use of vaccines is reasonable (*Konzentration, Grund, Einsatz, Entscheidung, Aufklärung, Nebenwirkungen*).

### Russian-Speaking Actors

#### Topic Structure

Explicitly expressed information, which makes up the topic structure of the Russian-language database, makes it possible to identify the following topics that are of greatest interest for users discussing the problems of COVID-19 after the commencement of the vaccination campaign:

• Effect of the vaccine in **humans** (*люди*- connection weight of 96; frequency of 36,332):

*Vector vaccines use viruses that are safe for humans and cannot reproduce in the human body (vectors).*

*This is the world’s first registered vector vaccine based on a new technological platform that involves human adenoviruses Ad26 and Ad5, which carry the S gene of the coronavirus protein. The vaccine is developed at the N. F. Gamaleya Center, Russia.*

• Features of various vaccines and their efficacy (**vaccine/*вакцина, вакцинация, прививка*:** connection weight of 82; frequency of 201,188; **vaccination:** connection weight of 75; frequency of 37,208):

*The efficacy of the Sputnik V vaccine of 91.4% was confirmed by the analysis of data at the final checkpoint of clinical trials*.

*Kryuchkov noted that cases of body temperature rise after vaccination against the coronavirus are not the only possible early post-vaccination manifestation*—*these can also include “injection site masses, as well as body temperature rise, general weakness, and malaise, that is, the classic manifestations of ARVI,” Sputnik radio reports.*

• Vaccination duration (**year/год** - connection weight of 79; frequency of 66,711; **time/время**: connection weight of 75; frequency of 19,702; **day/день**: connection weight of 71; frequency of 17,506):

*Vaccination against coronavirus at the level of 70% of the population in each country by the middle of next year will stop the pandemic and restart the global economy.*

*But such medieval obscurantists like you ensure the presence of COVID that already has a chance to achieve such mutations against which vaccines will show increasingly lower efficacy. Time and anti-vaxers work for COVID.*

• Significance and features of **vaccination** (connection weight of 76; frequency of 12,702):

*“If a person’s vaccination was, as they say, without a hitch, that is, without fever and so on, this doesn’t mean this person will not have immunity. On the other hand, it cannot be said that if a person has been in bed with a high temperature of 39 degrees for three days after their vaccination, then their immune response will definitely be higher,”* Kryuchkov explained.

• Efficacy and distribution of the vaccine **“SputnikV”** (connection weight of 73; frequency of 26,167):

*Curiously, this new situation provides arguments for both proponents and opponents of vaccinations. The first are convinced that vaccination should be even more massive (60% of those vaccinated, which were mentioned at first, are no longer enough), and that it is necessary to strengthen immunity with additional or repeated doses, since the common use of Sputnik V may not work.*

• Effect of vaccination on the spread of **COVID-19** (connection weight of 80; frequency of 30,533):

*In addition, it is noted that there is, currently, a global inequality in terms of access to vaccines against COVID-19, and, in poorer countries, far fewer people are fully vaccinated or received at least one dose than in richer countries, where vaccines are produced and governments have begun to talk about revaccination.*

• Public discussions of various issues related to vaccination, the formation of immunity, protection against the coronavirus infection (**question:** connection weight of 68; frequency of 18,403):

*My whole family was ill with this COVID; we’ve come through it easily, without pneumonia and side effects, only temperature was sometimes higher, and some of did not even notice that they had been ill; but the essential point about which everyone is silent is that: how long does the immunity last after the illness?*

• New cases, spread of the infection (**new:** connection weight of 68; frequency of 4,548; **cases:** Bec связи–68; frequency of 2,204):

*As of June 24, 2021, in Moscow, the situation with the new coronavirus COVID-19 remains tense. New cases of the coronavirus infection were recorded in various districts and cities of the region. The total number of cases in Moscow was 1,315,841 people.*

• Discussion of measures being taken to combat the pandemic, **work** on vaccination (**work:** connection weight of 70; frequency of 59,468):

*Then work began on obtaining virus isolates, and whole-genome sequencing was performed, as a result of which a vaccine strain was obtained and another strain that is used for quality control.*

#### Semantic Network

**FIGURE 7:**
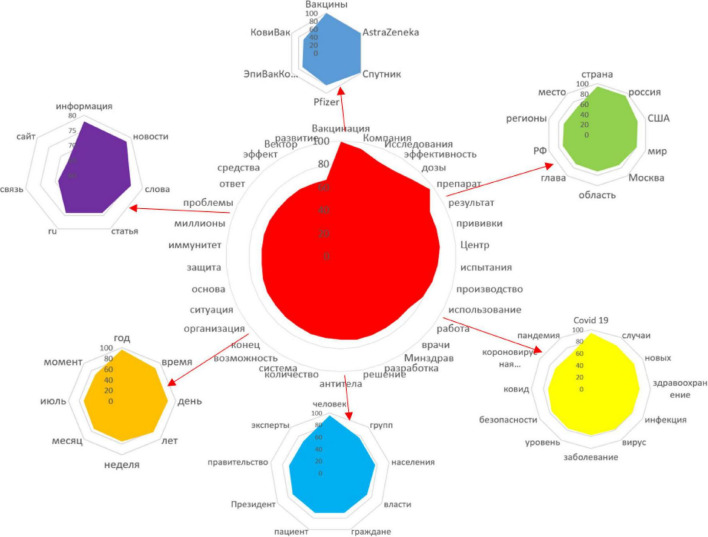
Features of vaccine production and specificity of vaccination.

Significant nominations of the semantic network with the following connection weights: vaccination (99), campaign (94), research (88), efficacy (87), doses (90), medication (96), result (86), vaccinations (86), center (86), trials (84), production (82), use (79), work (74), doctors (73), MOH (73), development (73), solution (73), antibodies (72), quantity (72), system (72), opportunity (71), end (71), organization (71), situation (71), basis (70), protection (69), immunity (69), millions (69), problems (69), response (68), means (68), effect (68), Vector (67), and development (67) (Figure 7).

Contexts are as follows:

*Why are Russians afraid of vaccination? Expert opinion. At the moment, Russia ranks number one among countries the population of which is skeptical about vaccination*.

*We already heard about it. Places for people with the right skin color, or a sign in the form of a yellow star on clothes, so that one can immediately see who is in front of them. Get injected and you will have more rights than those not injected.*

1. Discussion of various types of vaccines.

Significant nominations of the semantic network with the following connection weights: vaccines (100), AstraZeneka (99), Sputnik (99), Pfizer (81), EpiVacCorona (69), and CoviVac (67).

Contexts are as follows:

*As for this point, you need to be vaccinated, with a foreign vaccine, in an ideal scenario. But this is almost impossible. Of the Russian vaccines, the best of three evils is, obviously, CoviVac. A good thing cannot be called “Sputnik” in the 21st century.*

2. Spread of infection.

Significant nominations of the semantic network with the following connection weights: COVID (19), cases (85), new (84), healthcare (82), infection (80), virus (79), disease (78), level (77), safety (76), COVID (72), coronavirus infection (69), and pandemic (68).

Contexts are as follows:

*Stop talking nonsense. It is disgusting to read. If you want to be vaccinated*—*get vaccinated, if you do not, whatever, stop blaming people for deaths.*

3. Territorial features of the infection spread and vaccination.

Significant nominations of the semantic network with the following connection weights: country (95), Russia (94), United States (83), world (81), Moscow (73), region (72), chapter (71), RF (71), regions (69), and place (67).

Contexts are as follows:

*The chart shows that Russia and Australia are the most worrisome because of the number of such doubters. I do not know what is wrong on the smallest continent. But the Russian phenomenon seems to be clear. One thing distinguishes our country from all the global powers listed in the study*—*that is the lack of choice.*

4. Subjects of vaccination, persons responsible for vaccination.

Significant nominations of the semantic network with the following connection weights: human (96), groups (77), population (77), authorities (72), citizens (71), patient (71), President (71), government (69), and experts (69).

Contexts are as follows:

*And as for anti-vaccinators for ideological reasons, it is something of “artificially created natural selection.” Word up. If a person does not care about himself or herself, then we must not let him or her be careless about others. For instance, antimaskers do not want to understand that a mask is not their protection from others. On the contrary, the mask is the protection of others from a possible carrier.*

5. Time characteristics of vaccination.

Significant nominations of the semantic network with the following connection weights: year (96), time (87), day (86), years (83), week (76), month (74), July (72), and moment (70).

Contexts are as follows:

*Russian statistics cannot be trusted, at all. It will show as many as is necessary at the current moment. And much time has passed. The vaccine is usually tested for 5–7 years. Are there any data on side effects in 5–7 years? Or in 3 years? That is a rhetorical question.*

6. Information support of vaccination.

Significant nominations of the semantic network with the following connection weights: information (78), news (78), words (76), article (74), ru (74), link (69), and website (67).

Contexts are as follows:

*Coronavirus is a poorly understood disease, and the effect of vaccines under development is, accordingly, the same. Experts do not yet have answers to many questions, hence the wariness of people. We are doing all the testing during the pandemic; that is, all information is being collected today. Our task was to obtain such a dose, and a vaccination schedule that ensures the body’s immune response is as quick as possible.*

The semantic network analysis allows identification of semantic focuses that are of particular importance for actors and characterize the perception of the vaccination process and motivation when making a decision about vaccination. When discussing the specifics of vaccine production and the vaccination process, the actors highlight the issues of vaccine efficacy, the number of doses that will be delivered to various regions, as well as social problems, the possible infringement of the rights of people who reject vaccination. After the emergence of several types of vaccines, the actors began to actively discuss the merits of specific vaccines. AstraZeneka and Sputnik received the most attention in the discussion. Of the three Russian vaccines: Sputnik, EpiVacCorona, and CoviVac, the last one is recognized as the safest. The actors argued about the causes of the spread of the infection, severe consequences and deaths from the coronavirus infection, as well as by the state of modern healthcare in Russia. The persons responsible for the vaccination are criticized by both supporters of vaccination and opponents alike. The attention of users is attracted by the peculiarities of vaccination in different countries and in different regions of Russia, the readiness, and desire of the population to get vaccinated against the coronavirus infection. The actors are actively discussing the time characteristics of vaccination, as well as the time required to test a new vaccine and identify side effects. According to the analysis of the core of the semantic web, the actors negatively assessed the informational support of vaccination.

#### Associative Network

##### Stimulus Sputnik (10/108246)

**FIGURE 8:**
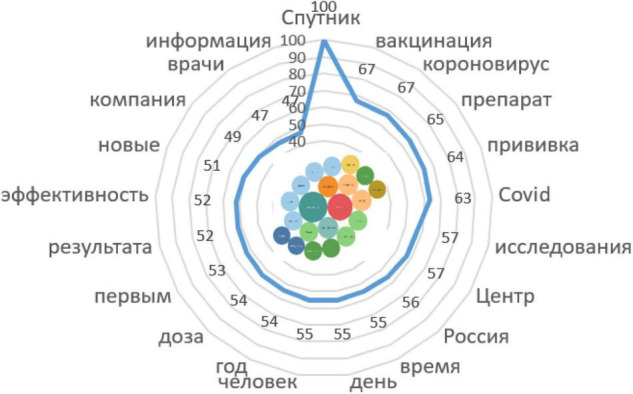
Associative network of the stimulus *Sputnik.*

Contexts with responses:

*Russia was the first country in the world to register a vaccine against COVID-19; it was Sputnik V, the medication developed by the N. F. Gamaleya Research Center for Epidemiology and Microbiology in cooperation with the Russian Direct Investment Fund* (Figure 8).

The vaccine is being produced; it arrives in the regions in batches, which is even good. The fact is that the Sputnik V vaccine requires special storage conditions.

##### Stimulus Risk Group (10/7163)

**FIGURE 9:**
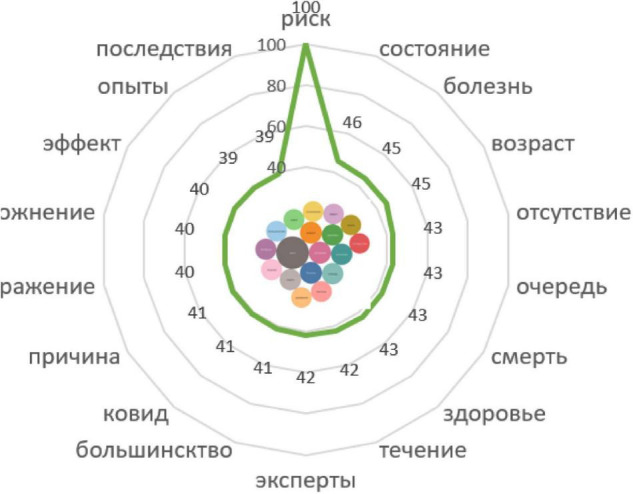
Associative network of the stimulus *Risk group.*

Contexts with responses:

*The risk groups include elderly people. In age groups over 70, vaccination reduces the risk of death among those affected by 31–52% (and the risk of getting affected by more than 90%)* (Figure 9).

After the emergence of the vaccine, Russian-speaking actors began to actively discuss the organization of vaccine production and vaccination campaigns, the consequences of the vaccine for various groups of the population, etc. Since the first vaccine was Sputnik V, this particular vaccine was in the spotlight. After the emergence of other vaccines, discussions began on the merits and demerits of each type, as well as the efficacy of vaccines against new strains.

The mandatory requirement for vaccination (at work or in educational institutions), the suspension from work of those who have not been vaccinated, the introduction of QR codes to visit public events, cafes, and restaurants, have caused significant criticism on social networks. Ironic stories about the inconsistency of the actions of the authorities and the conflicting statements have become widespread.

The Russian-speaking actors perceive sharply negatively the lack of choice of vaccines, since other Russian vaccines EpiVacCorona (10/13266) and CoviVac (10/11554) were not produced in the required quantities during this period, which formed a shortage and limited the choice of vaccines.

Data from associative networks also show that it is CoviVac that receives the most trust from users as the safest for health. Despite the publication of studies on the effectiveness and safety of the first Russian vaccine in official resources, the actors expressed opinions about the lack of sufficient time for testing, a list of contraindications, and fear of long-term consequences. The actors also believe that the developers of Russian vaccines have not found out whether a protective antibody titer is formed in the subjects, i.e., these vaccines may not be effective in preventing the coronavirus infection. In addition, mistrust is caused by information of posted instructions and on specialized resources.

The level of confidence in vaccines and the understanding of the need for vaccination are significantly undermined by the conflicting opinions of medical experts. The Internet interviews and statements of doctors who are skeptical about vaccination and declare the uselessness of vaccinations have become actively spread. These opinions are popular because they allow people who have doubts or are afraid of vaccination to receive external “authoritative” confirmation of their fears and justify their reluctance to get vaccinated.

The actors are convinced that foreign vaccines are more effective and safe in view of historically established and well-established stereotypes in public opinion about the advantages of imported products and drugs. The most actively discussed are AstraZeneca (10/4053) and Pfizer (10/4896). Moderna gets an index of (0/0) during associative search.

The actors express dissatisfaction with the lack of awareness of the consequences of vaccination for human health and think the issue is too politicized.

Particularly dangerous consequences of vaccination are recognized for people with poor health and chronic diseases, which are at risk (included in the risk group). Accordingly, people of elderly age groups automatically fall into risk groups.

The heightened emotional background of the vaccination discussion has led to the formation of digital conflictogenic zones. Vaccination supporters (including some actors who were forced to get vaccinated) sharply attack vaccination opponents (“COVID dissidents,” “covidiots”), accusing them of a threat to the life of society: *“anti-vaccine idiots get sick, infect others, die themselves, and threaten death to others, thereby causing damage not only to strangers but also to the state.”* Vaccination opponents insist on the right of choice, voluntariness of any medical procedures, on the right to be masters of their bodies and health.

## Discussion

Previous research on the perception and behavioral and psychological impact of COVID-19 was based on surveys; this study focuses on the analysis of content generated by social media users. The materials include both the opinions of actors who purposefully express their points of view and reasoning and messages that are related to various spheres of the actors’ lives, which indirectly relate to the pandemic and vaccination. The use of neural network technologies makes it possible to analyze not only implicit information but also explicit information to evaluate hidden reactions and assess actors that form the perception of the analyzed issues.

There is a large project COVID-19 IMPACT (see text footnote 1), which is an international online survey conducted in 78 countries/regions of the world studying the behavioral and psychological impact of COVID-19.

In particular, based on data from the COVID-19 IMPACT project, a study was conducted on the perception of COVID-19 in Europe at the early stage of the pandemic. Perception of a disease is an important predictor of emotional and behavioral responses in many diseases. The results of a large-scale study of IP addresses associated with COVID-19 across Europe have been published. The authors analyzed the temporal development, identified predictors (within demographics and contact with COVID-19), and examined the impacts of IP on perceived stress and preventive behaviors (Dias Neto et al., 2021).

This study analyzes the perception of the COVID-19 pandemic by Spanish-, German-, and Russian-speaking social media participants after the commencement of vaccination campaigns in the later stages of the pandemic using neural network technologies and psycholinguistic techniques, which made it possible to identify the implicit information and semantic focuses that are most important for the actors in Spanish-, German-, and Russian-speaking digital environment.

While before the emergence of any vaccine or treatment for COVID-19, all measures to contain and limit the spread of the infection depended on people’s behavior, after the commencement of the vaccination process, it became important to ensure that citizens positively perceive national vaccines, their safety, efficacy, and the power to stop the spread of the disease.

The results of this study are consistent with the findings of the study by Rosman et al. (2021) that the credibility of public health experts, scientists, and doctors who confirm the safety and efficacy of vaccines greatly contribute to the success of vaccination.

The results of our study also confirmed the conclusions by Rosman et al. (2021) that, for European countries, doubts about the safety of a vaccine expressed by official authorities significantly reduce the willingness of the population to get vaccinated. Thus, in March 2021, the safety of the AstraZeneca vaccine was questioned; several countries suspended its use. At the same time, politicians and public health experts were quick to reassure the public that all COVID-19 vaccines are safe and effective (Goldstein et al., 2020). Meanwhile, this no longer helped in terms of changing the attitude of the population, which began to increasingly lose faith in state institutions; a similar trend is intensifying, for example, in Germany since the beginning of 2021 (Betsch et al., 2021; Rosman et al., 2021).

This study correlates with the results of a comparative analysis of Chinese and American media reports on COVID-19 (Zhang et al., 2021), which reveal a close relationship of publicity coverage of the pandemic with political and ideological motives, and also confirm the importance of adhering to the principles of social responsibility.

Researchers have already noted the inconsistency of cultural comparisons of the disease perception (Bean et al., 2007; Kaptein et al., 2013). In the present study, a comparison was made with the inclusion of two European countries and a country outside Europe, thus changing the level of analysis from individual countries to European and non-European regions (north vs. south; west vs. east). Consideration of differences in the cultures and the severity of the pandemic in these countries, to some extent, contributed to the identification of differences.

The study of the peculiarities of the perception of the COVID-19 pandemic by Spanish-, German-, and Russian-speaking social media actors made it possible to identify differential and integral features of the topic structure, semantic, and associative networks built on the material of the three databases, which made it possible to determine the common and different characteristics of user perception after the commencement of the vaccination process and their attitude toward the vaccination itself.

Despite the coincidence of the topics identified in the topic structure that characterizes explicit knowledge of vaccination, the Russian-language topic network is more diverse. Russian-speaking users, as well as Spanish- and German-speaking ones, were concerned about such topics as the effect of the vaccine on people, the peculiarities of vaccination, the efficacy of the vaccines, and their influence on the further spread of COVID-19. The Russian- and Spanish-language databases show anxiety of the actors caused by the time characteristics of vaccination and new cases of the disease, which are less represented in the topics of the German-language discussions. Russian-speaking users are more actively discussing measures to combat the pandemic, work on organizing the vaccination process, immune development, and protection against the coronavirus infection. On the other hand, German-speaking users pay more attention to the territorial and age-related factors of vaccination, while Spanish-speaking users express a stronger fear of the disease.

Implicit knowledge revealed during the analysis of semantic networks made it possible to identify a large number of differential features characteristic of the perception of vaccination by speakers of the three analyzed languages. For German- and Russian-speaking users, it is important to have a well-adjusted production, efficiency, and response of the immune system to the vaccine, while Spanish users discuss rather the protective mechanism of the vaccine and the required number of doses, thereby confirming their absolute readiness for vaccination. The core of the semantic network reveals a strong concern of German users about the vaccine efficacy. The semantic emphasis highlighted in the German- and Russian-language materials shows that users are more concerned about the immune system’s response to the vaccine and its side effects on the body, while Spanish actors have a greater fear of the disease and death from COVID-19, and therefore ignore the possible consequences of vaccination.

Of key importance in the semantic network built on the material of all analyzed languages is the content on vaccines used in the respective country. Germany was a co-manufacturer of certain vaccines, while Spain did not have its own brand, and Spanish actors actively discussed all types of vaccines, including the Russian Sputnik, the possibility of which was not considered in Germany. The Russian actors discussed both Russian and foreign vaccines.

The further development of the pandemic and measures to prevent the spread of the infection are of concern to all users, but only German- and Spanish-speaking actors attach great importance to preventive measures complementary to vaccination. It should be noted that information about “complementary” measures was used in the official media in Germany and Spain as a means of pressure on vaccination opponents, since vaccinated citizens were exempted from their observance.

All three databases reflected the territorial strategy for combating COVID-19. For example, Germany and Spain align with EU countries, neighboring countries, and vaccine-producing countries; also, the competences on the national territory are differentiated, taking into account their specificity. The Spanish-language corpus also reflected data from Latin America, which can be explained by a linguistic proximity, and the Russian-language material often included discussions of the anti-COVID strategy of the United States considered a strong manufacturer and a long-term rival.

The content on vaccination subjects and those responsible for this process takes significant portions in all three databases. The main administrative structures and institutions involved in the organization of vaccination were also actively discussed. German-speaking users discussed groups of vaccinated persons in more detail, dividing them by age, gender, and the presence of pathologies. The upcoming elections in Germany have strongly influenced the vaccination debate, as comparisons of the anti-COVID policies of various leading parties have given the discussion a strong political dimension. The politicization of the topic of vaccination is also found in the Russian-speaking corpus.

The time factors of vaccination mostly coincided in all three language corpora and were associated with the chronology of the process organization in the respective countries.

The greatest differences were found in implicit knowledge devoted to information support. Open dissatisfaction with the anti-COVID policy in Germany was observed in the discussions of German-speaking users, who demanded respect for the foundations of democracy and respect for the opinions and choices of citizens during vaccination. The actions of vaccination opponents that were suppressed with the involvement of the police were actively discussed; and this caused an increase in protests and indignation. The study demonstrated the big availability and easy access to the big number of varied sources of information for the German users. The German-speaking actors also demanded to provide objective and truthful information about vaccination and its effectiveness and criticized the control and discrimination against vaccination opponents. The data show that the actors negatively perceive information support for vaccination, believe that there is a lack of convincing information in Germany, and doubt the advisability of vaccination. Negative perception is also noted among Russian-speaking users, who received a significant amount of conflicting information from various sources. Spanish-speaking actors mainly relied on official reports and data, without questioning the competence of the authorities in organizing vaccination campaigns. Thus, it can be concluded that the data indicate a high degree of loyalty of the Spanish actors to vaccination supported by trust in the government; therefore, the feasibility of vaccination was practically not discussed by the Spanish actors.

The German-speaking actors had wide access to information; an excess of information was perceived as a counterproductive factor in the vaccination campaign, as it led to increased doubts among residents about the need for a vaccine and the reliability of the information received, which, in the light of the upcoming elections to the Bundestag, acquired a political connotation and resulted in waves of protests.

The Russian-speaking users received information from various sources, but despite this, they demonstrate low readiness for vaccination. A rather common tactic in Russia is a “wait and see” attitude. Even the actors who believe vaccination is necessary to end the pandemic choose to delay vaccination for themselves and their family members out of fear of the consequences. The decisive factor in obtaining the vaccine is the requirements of employers and the administration of higher and secondary educational institutions, as well as the imposed bans on visiting cafes and restaurants without a QR code confirming vaccination.

Grave doubts of both the Russian and German actors are caused by fear of infringement of the rights of people who refuse to get vaccinated.

The Russian-speaking actors perceive negatively the mandatory requirement to get vaccinated (at work or to have the right to study) and the lack of choice of vaccines. Data show that it is CoviVac that receives the most trust from users as the safest for health. Despite the fact that the effect of the first Sputnik V vaccine has been studied in much more detail than the others, it causes a negative attitude among the actors.

The analysis of the content with responses to the stimuli vaccine and risk group in the three language datasets showed that the Russian actors trust foreign vaccines more than the Russian-made ones. A similar perception is characteristic for Spanish users. Meanwhile, the German actors doubt the high efficacy of the vaccine, especially against new virus variant; they are concerned about possible side effects and prefer to receive extensive information in advance and the opportunity to compare various types of vaccines and make a deliberate decision.

While people from risk groups in Spain and Germany have a reasonable priority in vaccination, Russian users consider the consequences of vaccination for people with poor health, chronic diseases, and those who are at risk, especially dangerous. It should be noted that, in Spain, teachers and medical workers were the first who were vaccinated as those included into the risk group.

In the Russian-speaking media space, strong emotions during the discussion of vaccination have led to the formation of digital conflictogenic zones. Vaccination supporters sharply attack vaccination opponents (“covid dissidents,” “covidiots”), accusing them of posing a threat to the life of society. Vaccination opponents insist on the right to be masters of their bodies and health. Similar opposition was observed among the German-speaking actors. The irreconcilability of positions has led to the emergence of a new conflictogenic digital zone also in the German-speaking cyber environment.

The most striking differential features of vaccination perception can be traced in the readiness to receive the vaccine: The Spanish-speaking actors show a positive attitude; they get vaccinated obediently and readily; the German-speaking actors perceive vaccination with suspicion and study the information thoroughly; the Russian-speaking users are characterized by “wait and see” attitude and fear of the consequences.

The results of the study confirm the official data on vaccinations (as of September 30): Spain—78%^7^; Germany—64%^8^; Russia—33%.^9^

## Conclusion

This study showed that, despite the similarity of the topic structures expressing explicit knowledge, the analysis of implicit knowledge revealed significant differences in the perception of the COVID-19 pandemic by Spanish-, German-, and Russian-speaking social media participants after the emergence of vaccines and the attitude toward vaccination itself. Different cultural features, the development of the pandemic, and social foundations in Germany, Spain, and Russia have led to a larger or lesser degree of readiness for vaccination in the population. With active campaigning to vaccinate in all countries, the Spanish-speaking users perceived vaccination as a salvation from COVID-19, protection from the infection, and a chance to overcome the fear of death; they trusted official sources of information and did not seek confirmation of the efficacy and safety of the vaccine in alternative sources. The German-speaking users thoroughly and critically studied the extensive amount of available information, were suspicious of new vaccines, and defended their right to choose, the foundations of democracy and freedom of opinion, which they also actively defended in offline actions and demonstrations. The Russian-speaking users received contradictory information from various sources; they demonstrate low readiness for vaccination and the fear of the consequences. A rather common tactic in Russia is a “wait and see” attitude.

The cognitive representation of the disease formed among the actors in the Spanish-, German-, and Russian-speaking media space, largely depended on the emotional and behavioral response of members of society. The peculiarities of perception of COVID-19 became one of the important factors in making a decision on vaccination and taking preventive measures for all three types of actors.

In addition, the results of the study confirmed the effectiveness of using multimodal neural network analysis to study speech perception in various discourses.

## Data Availability Statement

The original contributions presented in the study are included in the article/supplementary material, further inquiries can be directed to the corresponding author/s.

## Ethics Statement

The studies involving human participants were reviewed and approved by the Cyprus National Bioethics Committee (ref.: EEBK EΠ 2020.01.60). The patients/participants provided their written informed consent to participate in this study.

## Author Contributions

MP, AR, and OK performed the material preparation, data collection, analysis, and wrote the first draft of the manuscript. All authors commented on previous versions of the manuscript, read and approved the final manuscript, and contributed to the study conception and design.

## Conflict of Interest

The authors declare that the research was conducted in the absence of any commercial or financial relationships that could be construed as a potential conflict of interest.

## Publisher’s Note

All claims expressed in this article are solely those of the authors and do not necessarily represent those of their affiliated organizations, or those of the publisher, the editors and the reviewers. Any product that may be evaluated in this article, or claim that may be made by its manufacturer, is not guaranteed or endorsed by the publisher.

## Footnotes

1
https://ucy.ac.cy/acthealthy/en/covid-19-impact-survey

2
https://www.sketchengine.eu/

3
https://www.projectimplicit.net/

4
http://www.analyst.ru/index.php?lang=eng&dir=content/products/&id=ta

5
http://www.casos.cs.cmu.edu/projects/automap/

6
https://www.tableau.com/

7
https://www.mscbs.gob.es/profesionales/saludPublica/ccayes/alertasActual/nCov/documentos/Informe_GIV_comunicacion_20211001.pdf

8
https://ourworldindata.org/covid-vaccinations?country=DEU

9
https://gam-kovid-vak.ru/skolko-privito-ot-koronavirusa-v-rossii-na-4-oktyabrya/
